# Micro-CT evaluation of historical human skulls presenting signs of syphilitic infection

**DOI:** 10.1007/s00508-021-01832-z

**Published:** 2021-03-31

**Authors:** Sabine Fraberger, Martin Dockner, Eduard Winter, Michael Pretterklieber, Gerhard W. Weber, Maria Teschler-Nicola, Peter Pietschmann

**Affiliations:** 1 Institute of Pathophysiology and Allergy Research, Center for Pathophysiology, Infectiology and Immunology, Medical University Vienna (MUV), Vienna, Austria; 2 Core Facility for Micro-Computed Tomography, University of Vienna, Vienna, Austria; 3 Pathological-Anatomical Collection in de Fool’s Tower, Department of Anthropology, Museum of Natural History (NHM), Vienna, Austria; 4 Center for Anatomy and Cell Biology, Division of Anatomy, Medical University of Vienna (MUV), Vienna, Austria; 5 Department of Anthropology, Natural History Museum (NHM), Vienna, Austria; 6 Department of Evolutionary Anthropology, University of Vienna, Vienna, Austria

**Keywords:** Syphilis, Historical pathological-anatomical collection, Micro-computed tomography, Bone structure, Cortical bone alterations, Sclerotic reorganization, Treponema pallidum, Cortical porosity, Cortical thinning

## Abstract

**Background:**

In tertiary syphilis, *Treponema pallidum *triggers the formation of granulomatous nodules in various organs of the human body. Within the skeleton, predominantly in the skull and long bones, these characteristic syphilitic lesions cause typical patterns of bone damage. In this study, micro-computed tomography (µ-CT) was used to assess the microarchitecture of these osseous defects in untreated syphilitic skull bones.

**Material and methods:**

Bone structure of 30 macerated human skulls was noninvasively examined by means of µ-CT images (Viscom X8060 NDT). A total of 20 specimens showing typical morphological signs of syphilis were provided by the Collection of Anatomical Pathology of the Museum of Natural History in Vienna. They were compared to 10 macerated control skulls provided by the Division of Anatomy of the Medical University of Vienna.

**Results:**

All samples affected by syphilis showed perforating defects and increased porosity. Furthermore, we observed sclerotic reorganization and complete loss of the cortical bone in 80% of infected cases. Cortical thinning occurred in 75%.

**Conclusion:**

Our findings revealed extensive micromorphological bone destruction and a broad variability of osseous manifestations of (tertiary) syphilis.

## Introduction

Syphilis is a chronic, multistage sexually transmitted disease caused by the gram-negative spirochete *Treponema pallidum *[1]. Humans are the organism’s only natural reservoir and transmission occurs through direct contact with body fluids or sores of an infected individual, characteristically in sexual activities [2]. Untreated syphilis progresses in three stages: Spreading from localized primary lesions of the skin, *T. pallidum* induces a second phase of disease after 9–12 weeks. Thereafter, the microorganism persists in a latent stage for months or years. Dissemination throughout the whole body manifests as a tertiary stage, which is characterized by cardiovascular, neurologic, and deep cutaneous manifestations [3]. As a known complication of tertiary syphilis, destructive bone lesions occur but like other end-stage symptoms, have rarely been observed in their microstructural changes since the introduction of penicillin treatment in the 1940s.

Today, the general diagnosis of syphilis is based on clinical examination, serological tests and visualization of *T. pallidum* by dark-field microscopy or immunofluorescence. [4]. With respect to bone lesions that may appear as periosteal reactions in earlier manifestation stages, conventional X‑ray is widely used as a noninvasive tool of clinical investigation but solely provides two-dimensional macroscopic images. Histological analysis facilitates more detailed exploration but is laborious and destructive; hence, in clinical practice it is applied only in rare cases of diagnostic uncertainty. Medical computed tomography (CT) noninvasively delivers three-dimensional volume data; however, the resolution for studies of microstructures is limited. Thus, the micro-computed tomography (µ-CT) approach is the method of choice to cope with the requirement of getting three-dimensional images of bone architecture in high resolution and without any destruction of samples [5–7]. Using this technology, we aimed to shed light on the range of variation of microstructural changes at the calvaria of individuals who were infected by syphilis.

## Material and methods

### Skulls with alterations caused by syphilis

A total of 20 macerated dry skulls exhibiting signs of syphilis were included in this study. They were provided by the Pathological-anatomical Collection at the Fool’s Tower (PASiN), Natural History Museum Vienna. We selected skulls of individuals with reported clinical pathological diagnosis of syphilis that predate treatment by antibiotics and other medications to investigate the full extent of treponematous bone invasion. As Ehrlich and Hata introduced the arsenic-based drug salvarsan, the first effective treatment for syphilis in 1910 [8], individuals with a date of death before December 1909 were included; however, we cannot rule out that some patients might have received mercurial treatment or iodine therapy, which were used at that time and may have caused side effects on bone structure. A second determinant of selection was a minimum age of death of 25 years to eliminate the possibility of congenital syphilis. Our sample of 20 specimens that met these criteria comprised the skulls of 10 females (aged 27–65 years) and 10 males (age range 27–66 years) (Table 1).

**Table 1:** Sex and age of the syphilitic and control skulls

ID	Sex	Age (years)	Control	Sex	Age (years)
1	M	27	C1	M	66
2	M	40	C2	M	69
3	M	40	C3	M	76
4	M	42	C4	M	81
5	M	43	C5	M	89
6	M	43	C6	F	71
7	M	48	C7	F	72
8	M	52	C8	F	82
9	M	56	C9	F	90
10	M	66	C10	F	99
11	F	27	Mean age (years)		79,5
12	F	29			
13	F	31			
14	F	35			
15	F	53			
16	F	54			
17	F	58			
18	F	64			
19	F	65			
20	F	79			
Mean age (years)		47,6			

Skulls presenting signs of syphilitic infection derived from the Pathologic-anatomical Collection (PASiN), Natural History Museum Vienna. (N_males_ = 10; N_females_ = 10). The control skulls were kindly provided by the Division of Anatomy, Medical University of Vienna. (N_males_ = 5; N_females_ = 5).

Prior to µ-CT scans, each skull was macroscopically evaluated under fourfold magnification with respect to the diagnostic criteria of syphilis proposed by Ortner [9], documented and photographed. Thereafter, we scanned the complete skull to get an overall impression of the destructive force of syphilis. The permission for the analyses was granted by the ethical committee of the Medical University of Vienna (approval No:1260/2015).

### Control skulls

Additionally, 10 macerated dry skulls showing no macroscopically visible pathological changes were included for comparative reasons. They were provided by the Division of Anatomy, Medical University of Vienna. The control specimens were obtained from body donors who had consented to the use of their remains for research and teaching purposes. Absence of damages, clear layering of the calvaria, and an external table that appeared thicker than the internal one were considered as signs of healthiness. In order to obtain comparable data, maceration of the specimens was done in the same traditional way as in the affected skulls. Thus, they were macerated in 70 °C hot water without any additives [10, 11]. The sample consisted of 5 female (age 71–99 years) and 5 male skulls (age 66–89 years).

### Microcomputed tomography

As mentioned, µ-CT is an effective and nondestructive technique for imaging the microstructure of bones. To reduce the risk of movement artifacts, each sample was carefully positioned and fixed. Scanning of the skulls and three-dimensional reconstruction of raw data were carried out at the Vienna Micro-CT Laboratory, Core Facility for Micro-Computed Tomography, University of Vienna, with a Viscom (Viscom AG, Hannover, Germany) X8060 NDT scanner. Scans were performed with a source voltage of 130 kV, current of 330 µA, exposition time of 1400 ms, and zoom factor 1.6–1.7. Moreover, the µCT system was equipped with a copper filter (0.75 mm) for beam hardening reduction.

Based on our previous experience [12, 13], this study paid special attention to five alterations, i.e. cortical porosity, cortical thinning, cortical loss, sclerotic reorganization, and perforating defects. Cortical porosity was defined as increased number and size of pores in the cortical layer visible in µ-CT when compared to the control skulls. We used the term cortical thinning to refer to bone reduction of the internal or external table and cortical loss to describe the complete osteolysis of the internal or external lamina. Sclerotic bone is characterized by reorganization (pathological consolidation/compacting) of the microstructure of the laminae and/or diploe and is manifest by its radiopaque character. The abovementioned alterations observed in the syphilitic skulls (in comparison to the control skulls) were semi-quantitatively graded as follows: +: slight increase; ++: moderate increase; +++: severe increase. Finally, the presence of perforating defects was captured as the most serious bone alteration.

## Results

**Fig. 1:**
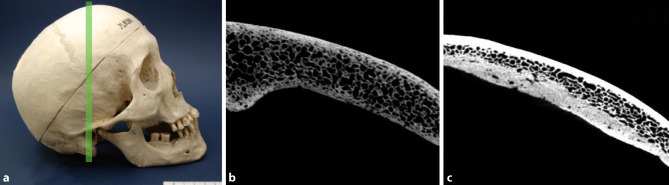
Cortical porosity. Skull of a 52-year-old man presenting with syphilis compared to a 66-year-old male skull without syphilitic signs used as a control. **a** Photograph of the syphilitic skull. The green line specifies the plane of the scan. **b** Cross-section of the affected frontal bone obtained by µ‑CT showing porous structure of both tables. **c** Skull bone of a 66-year-old man used as a control. Both tables are intact and clearly distinguished from the intervening diploe

**Fig. 2:**
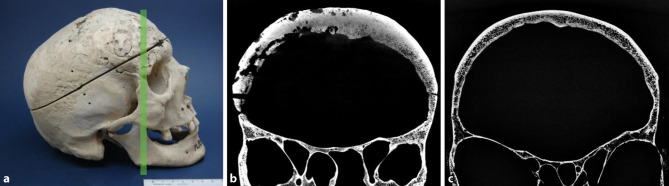
Sclerotic reorganization. Skull of a 43-year-old man presenting with syphilis compared to a 76-year-old male skull without syphilitic signs used as a control. **a** Photograph of the skull with macroscopically visible signs of syphilitic alterations. The green line indicates the plane of the scan. **b** Cross-section of the affected part of the skull obtained by µ‑CT showing lytic destructions and a massive sclerotic reorganization compared to the skull of the 76-year-old man in **c** lacking any infectious lesions used as control

**Fig. 3:**
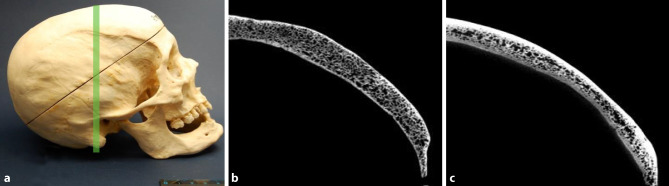
Cortical thinning. A 64-year-old female skull diagnosed with syphilis compared to a 72-year-old woman. **a** Lateral view of the syphilitic skull of a 72-year-old woman. The green line indicates the plane of the scan. **b** Micro-CT sectional image of the syphilitic skull bone showing remarkable thinning of the internal and external cortical bone compared to the control shown in Fig. 1c. **c** µ-CT cross-sectional image of a skull bone of a 72-year-old woman without any signs of infection used as a control

**Fig. 4:**
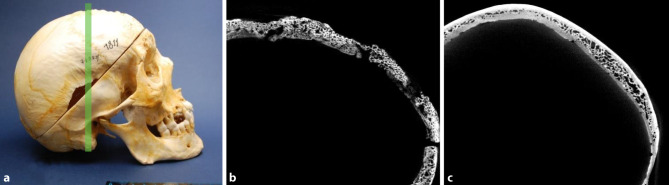
Cortical loss. Skull of a 69-year-old male diagnosed with syphilis and a 69-year-old male skull bone without syphilitic signs, used as a control. **a** Lateral view of the syphilitic skull. The green line again indicates the plane of the scan. **b** µ-CT scan of the syphilitic skull bone showing massive cortical loss compared to the compact cortical bone in Fig. 3c. **c** Skull of a 69-year-old man used as control without any signs of cortical bone loss

**Fig. 5:**
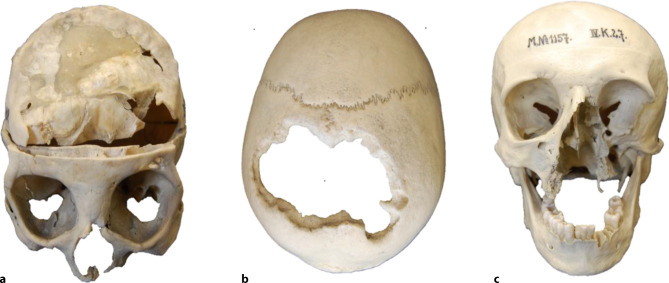
**a**–**c** Examples of tertiary syphilis:** a** frontal bone showing concave depression and impressive destruction (58-year-old woman).** b** Massive destruction of both parietal bones (52-year-old male). **c** Frontonasal syphilis showing extensive destruction of viscerocranium (56-year-old male)

In accordance with previous macroscopic reports [9, 14–25] and the duration of the infection our investigations revealed a great variation of pathological changes (Table 2, Figs. 1, 2, 3, 4 and 5). All affected samples showed at least one perforation (Fig. 5). The viscerocranium was affected in 80%, especially the hard palate and nasal septum. The neurocranium was involved in 50% of the individuals with syphilitic infection (Table 3).

**Table 2:** Changes in the microarchitecture of syphilitic skull bone

ID	Sex	Age (years)	Cortical porosity	Cortical thinning	Cortical loss	Sclerotic reorganization	Perforation
1	M	27	+		+		Y
2	M	40	++	++	+++	++	Y
3	M	40	+	+		+	Y
4	M	42	+			+++	Y
5	M	43	++	++	+++	+++	Y
6	M	43	+++	+++	++	+	Y
7	M	48	+			+	Y
8	M	52	+++	+	++	++	Y
9	M	56	+	++	+		Y
10	M	66	+++	++	+		Y
11	F	27	+		++	++	Y
12	F	29	++	+	++	+	Y
13	F	31	++	+	+	+++	Y
14	F	35	+++	++	+	++	Y
15	F	53	++	+++	++	+	Y
16	F	54	++			++	Y
17	F	58	+++	+++	+	+	Y
18	F	64	+++	+++	+		Y
19	F	65	++	++	++	+++	Y
20	F	65	+++	++	++	+	Y
Percentage of frequency [%]			100	75	80	80	100

+, ++, +++ semi-quantitative gradation of cortical porosity, cortical thinning, cortical loss and sclerotic reorganization by one single observer; *Y* yes. Frequency is calculated by including all graduations (+,++,+++)

**Table 3:** Evaluation of perforation defects

ID	Sex	Age (years)	Viscerocranium	Neurocranium
1	M	27	+	–
2	M	40	+	+
3	M	40	+	–
4	M	42	+	–
5	M	43	+	+
6	M	43	–	+
7	M	48	+	–
8	M	52	–	+
9	M	56	+	–
10	M	66	+	+
11	F	27	+	–
12	F	29	+	+
13	F	31	+	–
14	F	35	+	–
15	F	53	–	+
16	F	54	+	–
17	F	58	+	+
18	F	64	+	–
19	F	65	–	+
20	F	65	+	+
Percentage of frequency [%]			80	50

The analysis and the following comparison showed that in all affected individuals suffering from syphilis, increased porosity occurred (Table 2). For example, in the skull of a 52-year-old male presented in Fig. 1, this feature was pronounced in both the external and internal tables of the frontal bone. Fig. 1c depicts a skull of 66-year-old male lacking any infectious lesions used as a control.

Sclerotic reorganization was observed in 80% of the syphilitic skull bones. Fig. 2a, b shows a skull of a 43-year-old male showing a massive sclerotic reorganization in comparison to a 76-year-old male control skull (Fig. 2c). The internal and external tables, separated by the diploe, did not show any alterations and are clearly recognizable.

Our findings revealed that cortical thinning occurred in 75% of the syphilitic skulls. Fig. 3a, b shows a syphilitic skull of a 64-year-old female. The inner and outer tables are thin and their structure is irregular. Fig. 3c shows a skull of a 72-year-old woman without signs of cortical thinning.

Cortical loss appeared in 80% of infected cases (Table 2). Fig. 4 shows the high level of cortical loss in a skull of a 40-year-old man in comparison to that of a 69-year-old healthy male. Loss of one table was present in over 80% of the 16 specimens, while the skull of 2 males and 1 female showed loss of both the internal and the external tables. Interestingly, in about 75% of the 12 skulls that were affected by the loss of one cortical layer, the internal table was affected. A loss of the external table was found to be present only in one male and two females.

Hyperostosis frontalis interna was incidentally observed in the skulls of two females (recorded age at death 82 and 90 years, respectively) that were included in the control sample of noninfected individuals.

## Discussion

In this study for the first time a large number of skulls showing features of tertiary syphilis were analyzed by µ-CT. Our data reveal that extensive bone destruction and cortical porosity are the most prominent micromorphological features in syphilitic bone disease. Rühli et al. [26] compared the utility of µ-CT scanning to traditional histological sectioning for various osseous pathologies including one specimen of bone syphilis. They found that both techniques were of equal diagnostic value in general, which supports our methodological approach. Furthermore, Rühli et al. showed that changes of bone surfaces can be better visualized using µ-CT 3D images. In line with these findings, our study confirmed microcomputed tomography to provide detailed 3D images in a fast and nondestructive manner [28]. The aforementioned study [26] is the only comparative investigation of syphilitic bone using µ-CT. The authors showed cortical thickening of the skull bone, internally and externally, possibly a sign of bony remodeling. In contrast, our study demonstrated cortical porosity and bone loss of both cortical layers. The finding of Rühli et al. [26] may possibly imply the administration of antibiotics, because their study is based on cranial bone samples that were collected in the first half of the twentieth century [27].

Bone homeostasis is regulated by a delicate balance between osteoblastic bone formation and osteoclastic bone resorption [29]. In this complex process, different proteins play a key role: receptor activator of NF-κB ligand (RANKL) is a protein expressed by osteoblasts. It regulates the activity (formation, function and survival) of osteoclasts and the resorption of bone by interacting with its receptor activator of NF-κB (RANK), which is found on the surface of osteoclasts. Osteoprotegerin, another protein secreted by osteoblasts, is a natural inhibitor of RANKL and plays a role in regulating bone resorption. Macrophages express a variety of inflammatory cytokines and chemokines, such as interleukin‑1 (IL-1), IL‑2, tumor necrosis factor (TNF)-alpha and prostaglandin E [30]. Prostaglandin (PG) E upregulates RANKL and stimulates osteoclast formation, leading to osteolysis. Tp92, one of the surface antigens of *T. pallidum* could also be responsible for osteoclastogenesis. Jun et al. [31] showed that Tp92 homologs stimulated various factors involved in inflammation and osteoclastogenesis, such as IL-1ß, TNF-alpha, IL‑6 and PGE 2. Remarkably, several contemporary case reports impressively underline the massive bone destructive potential of (untreated) syphilis [32–39].

Beside extreme manifestations of osteoclast activity resulting in perforating defects the observed spectrum of osseous manifestations of syphilis also included cortical thinning and cortical osteoporosis. Perforating defects and cortical porosity was observed in all skulls and cortical thinning in three quarters of the skulls. Until now cortical osteoporosis has not been described in conjunction with syphilis. This finding also underlines the technical advantage of microcomputed tomography since the identification of cortical osteoporosis is beyond the resolution of conventional computed tomography or magnetic resonance imaging [32, 36]. Although cortical porosity is likely not specific for syphilis, this finding in conjunction with other features observed in our study could be helpful for the differential diagnosis of pathologies observed in human skeletal remains.

Not only osteoclasts but also osteoblast activities play an important role in the syphilitic remodeling process. Since syphilis is a relatively slowly developing disease, osteolytic destruction in many cases is followed by an osteoblastic reaction, which may lead to a pronounced thickening of the affected region [18]. As a likely manifestation of these osteoblastic reactions in our study sclerotic alterations were seen in the clear majority of cases (see Fig. 2b), i.e. in about 80% of the individuals.

Hyperostosis frontalis interna (HFI) involves the thickening of the frontal bone. It has been investigated by Western and Bekvalac [40] in historic female skeletal populations by microcomputed tomography. Consistent with our incident findings of HFI in two control skulls, Western and Bekvalac [40] showed that most cases of HFI are observed in older individuals.

There are some limitations that may influence our results. The documented diagnosis of syphilis in our 150-year-old skulls could neither be confirmed serologically nor microscopically, as we were limited to using macerated bones and nondestructive techniques. Furthermore, possible side effects of not documented mercurial treatment or iodine therapy cannot be ruled out. Nevertheless, due to our macroscopical evaluation with respect to the diagnostic criteria of syphilis proposed by Ortner [9] we could substantiate the selection of our samples. Lillie et al. [41] showed that there was significant reduction in cortical thickness with increasing age for females. This is in good agreement with our results. Our investigations showed that, even though the patients were younger than the controls, in the skulls of females suffering from syphilis both tables were significantly thinner than those of the control female samples.

Another important issue was the number of cases included in the given study. An extensive literature search yielded not a single comparable investigation. Therefore, for the planning of the study power calculations were not possible; however, in view of the sparsity of untreated syphilitic skulls, the descriptive nature of our study and lack of any similar works, we consider our results to be useful for others, particularly for researchers interested in palaeopathological subject matter.

## Conclusion

For the first time a considerable number of skulls exhibiting progressive alterations caused by an infection with *Treponema pallidum* from the preantibiotic era were analyzed by µ-CT. This nondestructive method revealed a broad variety of osseous manifestations of syphilis. In addition to severe osteolysis leading to perforating defects, also cortical porosis, cortical thinning and sclerotic alterations were detected.

Although the clinical manifestations of syphilis have changed over time, it is imperative that physicians are aware of the massive bone destructive potential of the disease.
